# Impact of Patient-Reported Allergies on Post-operative Complications and Healthcare Utilization Following Carpal Tunnel Release

**DOI:** 10.7759/cureus.53464

**Published:** 2024-02-02

**Authors:** Christopher Cheng, Oliver Dong, Kallie J Chen, Alexandre G Vesselle, Michael J Moses, Kyle J Chepla

**Affiliations:** 1 Orthopaedic Surgery, Case Western Reserve University, Cleveland, USA; 2 Orthopaedic Surgery, Case Western Reserve University School of Medicine, Cleveland, USA; 3 Orthopaedic Surgery, Cleveland Clinic, Cleveland, USA; 4 Plastic Surgery, MetroHealth Medical Center, Cleveland, USA

**Keywords:** clinical care outcomes, allergies, major depression disorder, complication of treatment, carpal tunnel release

## Abstract

Introduction: Open carpal tunnel release (O-CTR) is associated with high patient satisfaction and low complication rates. Risk factors for complications are well-established. Recent studies have found that patient-reported allergies (PRAs) and psychiatric comorbidities may be associated with increased complication rates. The impact of these factors after elective hand surgery has not been evaluated. This study sought to identify whether PRAs and psychiatric comorbidities are associated with complications after O-CTR and to evaluate their association with prolonged follow-up and the need for post-operative occupational therapy (OT).

Methods: Patient demographics, PRAs, Patient Health Questionnaire-2 score, Charlson Comorbidity Index, Carpal Tunnel Symptoms-6 score, postoperative complications, OT utilization, and time to final follow-up were recorded for patients who underwent elective O-CTR between 2014 and 2022. Multivariable binomial logistic regression analysis was used to determine pre-operative variables associated with increased risk for complication.

Results: About 250 patients met the inclusion criteria. Fifty-one (20.4%) patients developed minor complications, including scar tenderness (N=34, 13.6%), superficial wound dehiscence (N=9, 3.6%), and superficial infection (N=8, 3.2%). There were no major complications. Independent risk factors for complications included PRAs (OR 1.80, p<0.01) and PHQ-2 score (OR 1.39, p=0.04). Five or more PRAs and PHQ-2 score ≥3 are significant independent risk factors for increased post-operative complications. Increased PRAs and PHQ-2 scores were associated with longer follow-up (p=0.01 and p<0.01, respectively) but not increased OT utilization.

Conclusion: An increased number of PRAs and higher PHQ-2 scores are significant, independent risk factors for minor complications following O-CTR. Risk adjustment and peri-operative counseling should incorporate and account for these variables.

## Introduction

Open carpal tunnel release (O-CTR) is a commonly performed surgical procedure associated with high patient satisfaction scores and low complication rates. The reported re-operation rate after O-CTR for major complications ranges from 0.3% to 3% [1,2]. However, minor complications, including superficial infection and scar tenderness, are more common with a reported incidence of up to 36% [3-5]. Medical and demographic risk factors for major complications and revision surgery are well-established. However, recent studies of complication rates after spine surgery and total joint arthroplasty have found an additional association with patient-reported allergies (PRAs) [6-10]. Utilization of suboptimal perioperative antibiotic prophylaxis secondary to PRAs has been cited as a cause of post-operative infection in arthroplasty. The presence of multiple drug reactions has also been hypothesized in the immunology literature to have an overactive host immune response that decreases antimicrobial response [11]. Psychiatric comorbidities, either as an independent or co-dependent factor in conjunction with self-reported allergies, however, represent another potential influence, particularly in more subjective post-operative complications. In a population registry study, subjects with self-reported food and non-food allergies were at 1.8-fold greater odds of also having major depression or bipolar disorder [6]. The relationship between both PRAs and potential psychiatric underpinnings on post-operative complications has not been studied in the elective hand surgery setting [12].

Understanding this relationship is important as preoperative identification of patient-specific, psychosocial risk factors for post-operative complications could be used to identify patients who might benefit from additional peri-operative support. The purpose of the current study is to 1) identify whether PRAs and psychiatric comorbidities are associated with postsurgical complications after elective O-CTR and 2) determine whether PRAs and psychiatric comorbidities are associated with the need for post-operative occupational therapy (OT) and longer follow-up. We hypothesized that PRAs and depression are associated with a higher rate of complications, increased length of follow-up, and greater OT utilization after O-CTR. We hypothesize that PRAs and depression will exhibit a co-dependent influence on post-operative complications.

## Materials and methods

This study was approved by the MetroHealth Medical Center Institutional Review Board (STUDY00000256). A retrospective chart review was conducted for all patients who underwent elective, primary O-CTR by a single, fellowship-trained hand surgeon at a tertiary referral center between the years 2014 and 2022. O-CTR was performed in the operating room, with a single dose of pre-operative antibiotics, under local or monitored anesthesia care using the same surgical technique. Patient charts were identified by a search of current procedural terminology code 64721 (neuroplasty and/or transposition median nerve at the carpal tunnel). A total of 644 patients were identified on the initial database query. Study exclusion criteria included O-CTR completed as a sequela of trauma (72 patients), revision O-CTR (11 patients), presence of any concomitant elective surgery (43 patients), and patients younger than 18 years of age (16 patients). Patients who underwent O-CTR for bilateral disease were counted only once.

A priori power analysis identified a sample size of 243 patients necessary to provide 80% power to detect a five-fold increased re-operation rate following O-CTR. To satisfy this, 250 of the 502 patients who met the inclusion criteria were randomly selected using a number generator. Patient demographics, number of PRAs, Patient Health Questionnaire-2 (PHQ-2) score, Charlson Comorbidity Index (CCI), carpal tunnel symptoms-6 (CTS-6) score, electrodiagnostic testing (EDX), post-operative complications, frequency of OT utilization, and follow-up duration were recorded. CTS-6 scores were extracted from the provider’s initial hand clinic encounter note. The number of PRAs were identified in the electronic medical record, associated at the time of the initial visit. PHQ-2 values were obtained uniformly at their primary care visits, and the most recent value before the initial presentation in our clinic was recorded. Data recorded as part of the EDX included components of the nerve conduction study (sensory latency, motor latency, conduction velocity) and electromyography (recorded as the presence or absence of pathological end-organ electrical activity).

Complications were recorded as “minor” or “severe.” “Minor” complications were defined as those able to be managed non-operatively (including scar sensitivity, superficial dehiscence, and superficial infection) and required additional follow-up to monitor response to conservative intervention. In our practice, patients recovering from O-CTR are typically scheduled to be evaluated once, at approximately two weeks. At this visit, patients are uniformly and extensively counseled regarding the home, independent wound care, and scar massage, as well as expected sensory and motor recovery based on the severity of pre-operative nerve compression. The requirement for any additional in-office follow-up was used to delineate a “minor” complication (such as scar sensitivity) from an expected part of post-operative recovery. Follow-up for issues unrelated to the index surgery (such as ipsilateral hand pathology, contralateral CTS, etc.) was excluded from the final calculation of follow-up duration. Chart review was followed for up to six months post-operatively to monitor for complications. “Severe” complications were defined as those requiring a return to the OR. In addition to the total number of PRAs, data regarding the nature of the PRA including the category (environmental, food-based, antibiotic, analgesic, other) and reaction (anaphylactic or local) were recorded. The PHQ-2 score is a commonly utilized point-of-care depression screening instrument. A score greater than or equal to three is associated with major depression, with a reported sensitivity of 83% and specificity of 92% [13]. Psychiatric diagnoses present at the time of surgery were also recorded.

Standard descriptive statistics were used to describe the study population for patients with and without complications. After performing tests for normality, univariable analysis was used to compare baseline demographics, covariates, and outcomes between the two cohorts using student t-tests and Wilcoxon rank sum tests for parametric and nonparametric variables, respectively, and chi-squared and Fischer’s exact tests for categorical variables. Multivariable binomial logistic regression was used to determine the association between pre-operative variables and the presence of any post-operative complication. Similarly, multivariate linear regression was used to determine the association between pre-operative variables and the number of post-operative OT visits and duration of office follow-up. The presence and strength of multicollinearity between each independent variable were assessed using variable inflation factors (VIF). To further characterize the relationship between the number of PRAs and post-operative complications, post-hoc testing following contingency analysis was performed using the Bonferroni adjustment for multiple testing (α=0.015). A p-value <0.05 was considered significant in all other tests. Descriptive statistics were expressed as means and standard deviation and medians and interquartile ranges for parametric and nonparametric continuous variables, respectively, and frequencies and percentages for categorical variables.

## Results

A total of 250 patients who met the inclusion criteria were included in the study. A summary of baseline demographics and clinical characteristics between patients with and without complications is shown in Table 1.

**Table 1:** Univariate analysis of baseline demographics and covariates between cohorts with and without complication following O-CTR N: number; SD: Standard deviation; IQR: Interquartile range; CTS-6: Carpal tunnel syndrome-6 test; EDX: Electrodiagnostic testing; PHQ-2: Patient Health Questionnaire-2; PRA: Patient-reported allergies; O-CTR: Open carpal tunnel release

Variable	No complication	With complication	p-value
Number (n) of patients	199	51	
Age, mean (SD)	54.6 (13.9)	56.2 (11.9)	0.44
Sex			0.10
Female	137 (68.8%)	41 (80.4%)	
Male	62 (31.2%)	10 (19.6%)	
BMI, median (IQR)	30.9 (27.4, 35.8)	29.9 (25.8, 35.9)	0.47
Laterality			0.09
Left	93 (46.7%)	17 (33.3%)	
Right	106 (53.3%)	34 (66.7%)	
Hand dominance			0.46
Left	25 (12.6%)	4 (7.8%)	
Right	174 (87.4%)	47 (92.2%)	
Tobacco status			0.39
Smoker	152 (76.4%)	36 (70.6%)	
Non-smoker	47 (23.6%)	15 (29.4%)	
CTS-6, median (IQR)	16.5 (13.0, 16.5)	16.5 (16.5, 16.5)	0.30
Patients with EDX, n (%)	157 (78.9%)	36 (70.6%)	0.21
EDX components, mean (SD)			
Sensory latency (ms)	4.1 (3.0, 7.7)	3.9 (3.0, 5.1)	0.35
Motor latency (ms)	5.1 (4.6, 6.5)	4.9 (3.8, 7.2)	0.60
Sensory conduction velocity (m/s)	35.8 (31.0, 42.0)	45.3 (30.8, 48.5)	0.047
Pathologic electric activity present, n (%)	78 (84.8%)	14 (15.2%)	0.30
Charlson Comorbidity Index, median (IQR)	1.0 (0.0, 3.0)	2.0 (1.0, 3.0)	0.29
PHQ-2, n (%)			<0.01
Less than 3	178 (89.4%)	38 (74.5%)	
3 to 6	21 (10.6%)	13 (25.5%)	
Number of PRAs, n (%)			<0.01
Less than 2	122 (61.3%)	21 (41.2%)	
2 to 5	63 (31.7%)	16 (31.4%)	
5 or more	14 (7.0%)	14 (27.5%)	
PRAs by category, n (%)			0.54
Environmental	38 (12.1%)	19 (11.8%)	
Food-based	24 (7.7%)	16 (9.9%)	
Antibiotic	87 (27.8%)	41 (25.5%)	
Pain meds	63 (20.1%)	41 (25.5%)	
Other	101 (32.3%)	44 (27.3%)	
PRA by reaction, n (%)			0.41
No. of anaphylactic reactions	19 (6.1%)	13 (8.1%)	
No. of minor/local reactions	294 (93.9%)	148 (91.9%)	
No. OT visits, median (IQR)	0.0 (0.0, 0.0)	0.0 (0.0, 1.0)	<0.01
Follow-up duration (weeks), median (IQR)	2.3 (2.0, 7.3)	8.1 (6.1, 17.1)	<0.01

There were no significant differences in patient age, sex, BMI, hand dominance and operative laterality, smoking status, pre-operative CTS-6 score, and CCI. There were no major complications necessitating a return to the OR. Fifty-one (20.4%) patients had minor complications, including 34 (13.6%) with scar tenderness requiring additional follow-up, 9 (3.6%) with superficial wound dehiscence managed successfully with local wound care, and 8 (3.2%) with superficial infection treated with oral antibiotics. PHQ-2, CTS-6, and CCI scores were available for all patients. Forty-eight (19.2%) patients had a previous diagnosis of major depressive disorder and 86 (34.4%) had a previous psychiatric diagnosis. EDX was available for 193 (77.2%) patients. Regression analyses involving EDX were conducted separately including only patients with available data. The most recent EDX results were available on average 9.0 ± 14.2 months prior to the date of surgery.

Univariate analysis revealed significant differences in PHQ-2 scores (p<0.01), number of PRAs (p<0.01), and sensory conduction velocity (p=0.047) between cohorts with and without complication (Table 1). Multivariate analysis, however, revealed that PRAs (OR: 1.80, 95% CI: 1.18-2.75, p<0.01) and PHQ-2 score (OR: 1.39, 95% CI: 1.07, 1.99, p=0.04) were the only independent risk factors for post-operative complication (Table 2).

**Table 2:** Multivariate analysis of pre-operative risk factors for complications following O-CTR PHQ-2: Patient Health Questionnaire-2; CTS-6: Carpal Tunnel Syndrome-6 test; NCS: Nerve conduction studies; EMG: Electromyography; O-CTR: Open carpal tunnel release

Variable	Odds ratio	95% confidence interval	p-value
Age (years)	1.04	0.95-1.14	0.36
Symptom duration (months)	0.99	0.96-1.02	0.38
Body mass index	0.92	0.81-1.04	0.16
Tobacco use	1.06	0.23-4.94	0.95
Sex	0.98	0.16-6.04	0.98
PHQ-2 score	1.39	1.07-1.99	0.04
CTS-6 score	1.08	0.91-1.29	0.38
Charlson Comorbidity Index	1.10	0.64-1.92	0.73
Patient-reported allergies	1.80	1.18-2.75	<0.01
NCS - sensory latency	1.04	0.49-2.17	0.93
NCS - motor latency	1.15	0.49-2.72	0.75
NCS - conduction velocity	1.05	0.96-1.15	0.26
EMG (end-organ abnormality)	4.11	0.76-22.26	0.10

**Figure 1:**
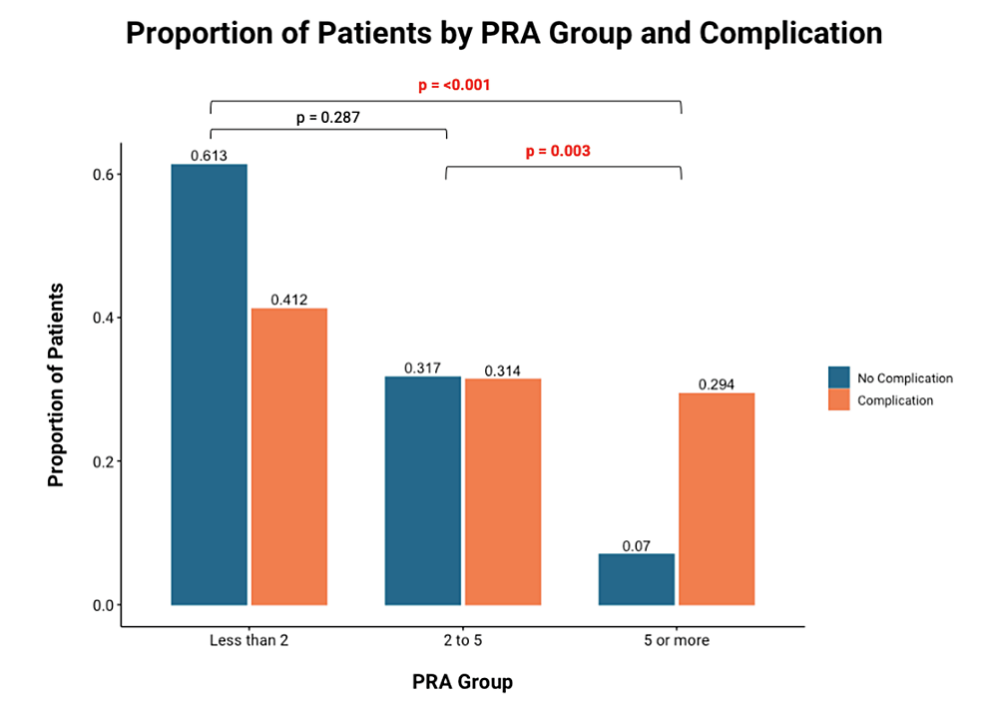
Bonferroni-corrected univariate chi-square analysis of the correlation between the number of PRAs and complication Significance* *p<0.05 PRA: Patient-reported allergies

The previous diagnosis of major depressive disorder was not an independent risk factor for post-operative complications. Collinearity, or the presence of co-dependent association within independent variables between PRA and PHQ-2 was not found (VIF of 1.17 and 1.11, respectively) within this multivariate analysis. Subgroup multivariate analysis of each post-operative complication revealed PRA as a statistically significant risk factor for post-operative scar sensitivity only (OR: 2.52, 95% CI: 1.16, 5.46, p=0.02). On post-hoc subgroup analysis, the presence of five or more PRAs was significantly correlated with post-operative complications (Figure 1).

**Figure 2:**
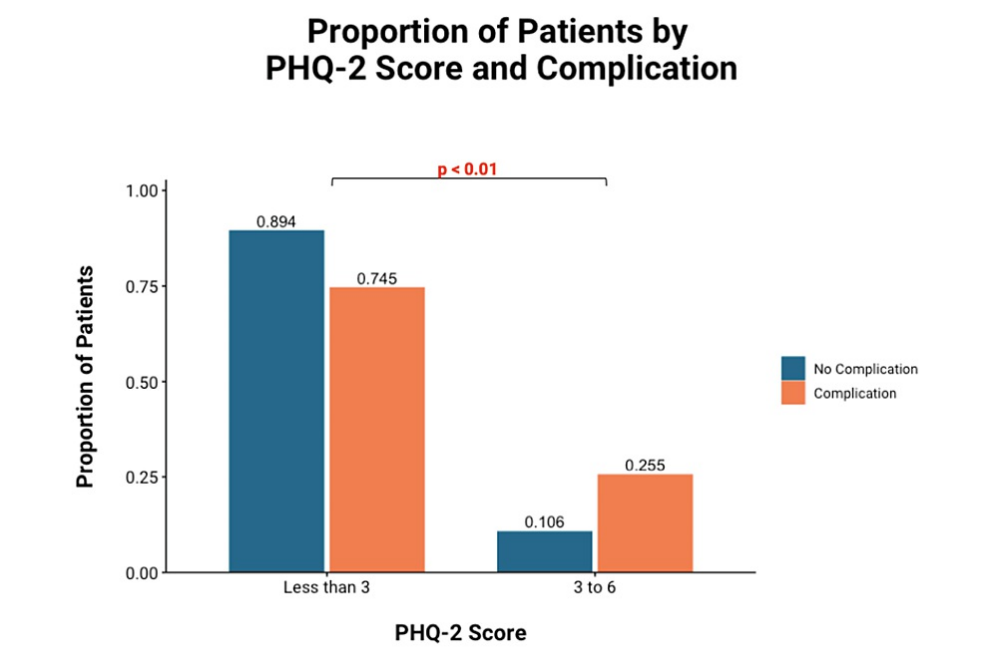
Bonferroni-corrected univariate chi-square analysis of the correlation between the PHQ-2 score and complication Significance p<0.05 PHQ-2: Patient Health Questionnaire-2

No significant difference was identified between patients with fewer than two and patients with two to five PRAs. Similarly, patients with PHQ-2 scores ≥3 were significantly correlated with post-operative complications compared to those with scores less than three (Figure 2).

The PHQ-2, as a point-of-care depression screening tool, confirmed current depression in patients with a previous diagnosis of major depression in 11 patients (22.9%), did not identify current depression in 37 patients (77.1%), and identified 22 patients as having concerning depressive symptoms without a previous formal diagnosis.

Assessment of the same pre-operative risk factors on office follow-up duration and OT utilization revealed increased allergies and PHQ-2 to be associated with longer office follow-up duration after surgery (p=0.01 and p<0.01, respectively) but not with a significant increase in OT utilization (p=0.62 and p=0.72, respectively).

## Discussion

Medical comorbidities have frequently been cited as major risk factors for complications following surgery [14-16]. Recently in the arthroplasty literature however reports have identified PRAs, not medical comorbidities, to be independently associated with poorer post-operative functional scores [17]. Fisher et al. reported that PRAs independently predicted the need for revision for infection in hip and knee arthroplasty. In their study, each additional PRA increased the infection risk by 1.11 times [9]. Elrick et al., in their retrospective review of 411 primary shoulder arthroplasties, found PRAs to be a statistically significant and stronger predictor of poor post-operative QuickDASH score when compared to the Charlson Comorbidity Index [10]. In their post-hoc analysis, patients with ≥2 PRAs were also found to have significantly worse post-operative QuickDASH scores.

PRAs have also been found to be associated with increased opioid consumption post-operatively and concomitant depressive symptoms [6,18]. The impact of depression on post-surgical outcomes is likely unappreciated by surgeons. A recent analysis of over 450,000 Medicare patients who underwent O-CTR found that depression had a greater odds (OR 1.78) of resulting in post-operative infection than any medical comorbidity [16]. Given these findings, the current study was designed to evaluate whether PRAs and PHQ-2 scores are associated with an increased risk of complication after elective hand surgery.

Our retrospective review identified no major complications and minor complications consisted primarily of peri-incisional pain and superficial infection. Interestingly, no significant differences were seen between age, sex, gender, smoking status, and medical comorbidities (CCI), all of which are variables previously associated with poor outcomes following O-CTR [16]. A possible explanation for this may relate to our inclusion of all complications, rather than infection alone. It should also be noted that the majority of complications recorded in our cohort involved persistent peri-incisional pain. In non-heavy laborers, the exact etiology for this is often unclear [5]. The more subjective nature of complications such as scar sensitivity may not correlate with a particular medical comorbidity, rather this study provides support for a possible psychological association.

Similar to previous work, univariate analysis revealed significant differences in PHQ-2 scores (p<0.01), number of PRAs (p<0.01), and sensory conduction velocity (p=0.048). However, when a multivariate model was used it was found that pre-operative PRAs and PHQ-2 score were the sole independent risk factors for post-operative complications. On subsequent univariate analysis, it was identified that patients with five or more PRAs were found to be at significantly higher risk for complications compared to those with fewer than five PRAs. Similarly, patients with a PHQ-2 score ≥3 were also at higher risk for complications than counterparts with scores less than three. Thresholds of five PRAs and PHQ-2 ≥3 can thus be established as risk factors for post-operative complications. Finally, increased PRAs and PHQ-2 scores were associated with longer follow-up times, but neither was associated with an increased need for post-operative OT.

Limitations

This study has several limitations in addition to its retrospective design. First and most importantly, the correlations established as part of the multivariate and univariate analyses do not imply causation. The true cause of complications following O-CTR is likely multifactorial. In fact, the absence of strong collinearity between PRAs and PHQ-2 found in this study suggests that even between these two variables, a dependent relationship cannot be drawn. Each variable likely exerts an independent effect on complications following O-CTR. It is noted that PRAs and PHQ-2 are variables that often cannot be modified by the treating surgeon, but may be helpful in identifying possible “at-risk” patients. It is interesting to note that PHQ-2 scores ≥3, which is a point-of-care reflection of current depression symptoms, but not a prior history of depression or psychiatric disorder was found to be an independent predictor of post-operative complications. Furthermore, in the present study, 22 patients were identified by PHQ-2 to be at risk for depression without any previous diagnosis. This represents a gap in wholistic patient care. Review of this data point prior to surgery, with appropriate referral back to the patient’s primary care provider or psychiatrist may improve pre-operative optimization and post-operative outcomes. Further research will be required to identify whether pre-operative patient counseling or other methods of risk adjustment can reduce the risk of complications. Patient-reported, functional outcome metrics were not available for the current study to better elucidate relationships between the impact of the study variables and minor complications on overall patient satisfaction and function after O-CTR. Longer follow-up durations were identified in patients with higher PRA and PHQ-2, though the costs associated with this increased healthcare utilization were not able to be examined in the present study. The cohort in the present study, which is comprised of patients from a single surgeon's practice at a single institution, may also limit the generalizability of the current findings to different regions with different patient demographics. The current study, while made up of a relatively small number of patients, was powered to detect differences in re-operation but may have been underpowered to detect differences in secondary outcomes. However, post-hoc power analysis utilizing the rate of minor complications identified in the current study (20.4%), identified a sample size of 114 patients to provide 80% power to detect a two-fold increase in minor complication rate. As such, the study sample size was maintained per a priori power analysis. Finally, it is noted that only PRA was found to be a significant risk factor for post-operative scar sensitivity on subgroup analysis. Small sample sizes amongst the minor complications however limit conclusions that may be drawn regarding the relative impact of PRA and PHQ-2 on each complication type. Larger sample sizes will be required to adequately parse out these relationships.

## Conclusions

The findings of this study aim to provide a more holistic perspective on risk factors for complications following O-CTR, shedding light on the role that PRAs and PHQ-2 scores play in the post-operative course following O-CTR. We have shown that an increased number of PRAs and higher PHQ-2 scores are significant, independent risk factors for minor complications following O-CTR. Risk adjustment and peri-operative counseling should incorporate and account for these variables as markers of psychological well-being, and in addition to traditional medical optimization, may serve as an important opportunity to improve patient outcomes. Although further research is required to identify whether potential interventions such as psychological counseling or ensuring medication adherence could mitigate these risks, the findings in this study may assist surgeons in the identification of patients at greater risk for complications and can guide patient counseling, risk adjustment, and psychosocial components of patient-centered care.
